# Changes in Early T-Cell Subsets and Their Impact on Prognosis in Patients with Sepsis: A Single-Center Retrospective Study

**DOI:** 10.1155/2023/1688385

**Published:** 2023-12-26

**Authors:** Yanhua Li, Youquan Wang, Bin Chen, Jianxing Guo, Dong Zhang

**Affiliations:** ^1^Department of Critical Care Medicine, The First Hospital of Jilin University, Changchun, China; ^2^Department of Nephrology, The First Hospital of Jilin University, Changchun, China

## Abstract

**Objective:**

To analyze the early changes in CD3^+^, CD4^+^, and CD8^+^T-cell subset counts in patients with sepsis and their correlation with prognosis to provide a feasible basis for clinical immunomodulation in sepsis.

**Methods:**

This is a single-center retrospective study. The study enrolled sepsis patients (meeting SEPSIS 3.0 definition) who were admitted to the Department of Intensive Care Unit at the First Hospital of Jilin University from July 5th, 2018 to December 5th, 2019 and were aged 18 years or above. In addition, these patients underwent cellular immune testing (CD3^+^, CD4^+^, CD8^+^ T lymphocyte counts, and CD4^+^/CD8^+^ ratio) within 24 hours of ICU admission. Patient's clinical data including age, gender, infection site, APACHE II score, SOFA score, length of ICU stay, mechanical ventilation time, ICU mortality, 28-day mortality, and 3-year survival status were collected. The prognostic indicators and survival of the decreased and nondecreased groups of different subsets of T lymphocyte counts and CD4^+^/CD8^+^ ratio were compared.

**Results:**

A total of 206 patients were enrolled, with 76.7% having a decrease in CD3^+^ T lymphocyte count, 76.7% having a decrease in CD4^+^ T lymphocyte count, and 63.6% having a decrease in CD8^+^ T lymphocyte count. Furthermore, 21.8% had a lower CD4^+^/CD8^+^ ratio. Analysis showed that the CD3^+^ T lymphocyte count decreased group had a longer length of ICU stay [11 d (4, 21) vs. 7 d (4, 17), *P*=0.03], increased percentage of mechanical ventilation (67.5% vs. 51.0%, *P*=0.04), and extended mechanical ventilation time [144 h (48, 360) vs. 96 h (48, 144), *P*=0.04] compared to the nondecreased group. The 28-day mortality was higher in the decreased group of CD4^+^/CD8^+^ ratio compared to the nondecreased group (33.3% vs. 25.5%, *P*=0.29); however, the difference did not reach statistical significance. Logistic regression analysis revealed no significant correlation between the decrease in CD4^+^/CD8^+^ ratio and 28-day mortality (*P*=0.11). The 3-year follow-up revealed that the CD4^+^/CD8^+^ decreased group had a lower survival rate than the nondecreased group (33.3% vs. 53.4%, *P*=0.01).

**Conclusions:**

In the early stage of sepsis, most patients showed a decrease in CD3^+^, CD4^+^, and CD8^+^T-cell subsets, as well as in the CD4^+^/CD8^+^ ratio. The decrease in CD3^+^ and CD4^+^/CD8^+^ was related to some poor prognosis.

## 1. Introduction

Sepsis is one of the main causes of death among intensive care unit (ICU) patients. It has been reported to affect approximately 50 million cases worldwide annually [1]. Despite the increasing clinical adoption of sepsis management guidelines in recent years [2, 3], the mortality of sepsis shock remains high, exceeding 30% [4]. Moreover, sepsis survivors face a significantly higher risk of rehospitalization and long-term mortality compared to nonsepsis patients [5, 6]. Research has shown that 50% of sepsis survivors experience recovery upon discharge, while one-third of patients die in the second year, and one-sixth suffer from severe and persistent impairments, with mortality of 40%–80% in the subsequent three years [7, 8]. Therefore, improving the prognosis of sepsis patients and enhancing their long-term survival have become a focal point in current sepsis research. However, the pathogenesis of sepsis and the mechanisms contributing to increased long-term mortality are not yet completely elucidated, which in turn affects the selection and exploration of clinical treatment methods [9]. Numerous studies have shown that immune dysfunction plays a significant role in the occurrence and progression of sepsis [10, 11]. Immune dysfunction is likely one of the important factors influencing clinical outcomes and is closely associated with poor prognosis [12–16]. Therefore, early identification of changes in the immune status of sepsis patients is of utmost importance for the timely implementation of immunomodulatory therapy.

Therefore, this study focuses on the detection of major phenotypic counts of T lymphocytes, which have been clinically carried out in our hospital in recent years. We aim to summarize and analyze the early characteristics of CD3^+^, CD4^+^, and CD8^+^ T lymphocyte counts, CD4^+^/CD8^+^ ratio changes in sepsis patients, and their correlations with short-term prognosis and long-term survival rates. The findings aim to provide routine monitoring methods for immune status in sepsis patients at primary healthcare facilities and serve as a basis for clinical immunomodulatory therapy for sepsis patients.

## 2. Participants and Methods

### 2.1. Participants

This is a single-center retrospective study. Inclusion criteria are as follows: ① the patients with sepsis admitted to the ICU of the First Hospital of Jilin University from July 5th, 2018 to December 5th, 2019 who met the definition of SEPSIS 3.0 [17], showing clear infection foci and a SOFA score of ≥2 points; ② the patients aged 18 years or above; ③ the patients underwent cellular immune testing (CD3^+^, CD4^+^, CD8^+^ T lymphocyte counts, and CD4^+^/CD8^+^ ratio) within 24 hours of ICU admission.

Exclusion criteria are as follows: ① patients who refused treatment; ② patients who died within 24 hours after admission; ③ patients who are long-term users of immunosuppressants; ④ patients who are HIV-positive; ⑤ patients with incomplete data or failed follow-up.

This study has been approved by the Ethics Committee of the First Hospital of Jilin University (Ethics Approval Number: AF-IRB-036-01) and has obtained an exemption from informed consent.

### 2.2. Research Methods

#### 2.2.1. Clinical Data Collection

The levels of CD3^+^, CD4^+^, CD8^+^, and CD4^+^/CD8^+^ were detected by BD FACSCalibur CANTO flow cytometry within 24 hours after admission to the ICU. In addition, the age, gender, APACHE II score, SOFA score, infection site, shock occurrence, mechanical ventilation rates, mechanical ventilation time, platelet, procalcitonin, ICU length of stay, ICU mortality, 28-day mortality, 1-year mortality, and the survival status of 3-year follow-up were collected.

#### 2.2.2. Grouping Methods

The patients included in the study were divided into decreased group and nondecreased group of CD3^+^ cell count, CD4^+^ cell count, CD8^+^ cell count, and CD4^+^/CD8^+^ ratio based on whether their count of CD3^+^, CD4^+^, CD8^+^, and CD4^+^/CD8^+^ was lower than the normal range in our hospital. The reference value range was CD3^+^ (1012–2428/*µ*l), CD4^+^ (540–1224/*µ*L), CD8^+^ (312–1060/*µ*L), and CD4^+^/CD8^+^ (0.91–2.43).

#### 2.2.3. Observation Indicators

The primary observation indicator is 28-day mortality. Secondary observation indicators include the length of ICU stay, the proportion of patients receiving mechanical ventilation, the duration of mechanical ventilation, the incidence rate of ventilator-associated pneumonia, ICU mortality, 1-year mortality, and the 3-year survival status.

### 2.3. Statistical Methods

Statistical analysis was performed using SPSS 24.0 software. Normally distributed measurement data were represented as mean ± standard deviation (SD), nonnormally distributed measurement data were represented as median (interquartile range), and counting data were represented as a percentage. The homogeneity test of variance was performed for normally distributed measurement data, and the *t*-test was performed for comparison of mean values between two groups of normally distributed data with uneven variance. The *t*-test was performed for comparison of mean values between two groups conforming to normal distribution but with uneven variance, and the rank sum test was performed for measurement data conforming to nonnormal distribution. Comparison between the two groups was analyzed by Pearson's chi-square test. The Spearman correlation coefficient and variance inflation factor were performed to detect collinearity among the variables. Multivariate logistic regression analysis was performed on the risk factors that may affect the 28-day mortality of sepsis patients. Kaplan–Meier survival curves of enrolled patients within 3 years were plotted, and the log-rank test was performed. A *P* value less than or equal to 0.05 was considered statistically significant.

## 3. Results

### 3.1. General Data Statistics of Enrolled Patients

A total of 206 patients were included in this study.  Table 1 presents the age, gender, APACHE II score, SOFA score, and prognosis of the enrolled patients.

### 3.2. Characteristics of T-Cell Count Changes in Sepsis Patients

The count of CD3^+^, CD4^+^, and CD8^+^ T cells and the ratio of CD4^+^/CD8^+^ in sepsis patients within 24 hours after admission to the ICU showed that most of the 206 enrolled patients had decreased T lymphocyte counts of CD3^+^, CD4^+^, and CD8^+^, and 45 patients (21.8%) had a decreased CD4^+^/CD8^+^ ratio. Only 6 patients (2.9%) had an increased CD3^+^ T lymphocyte count. Of these 6 patients, two patients were diagnosed with malignant tumors and had increased CD4^+^ and CD8^+^ T lymphocyte count, while one patient had autoimmune disease with an increased CD4^+^ T lymphocyte count.  Table 2 provides a detailed breakdown of these results.

### 3.3. Correlation Analysis between Decreased T-Cell Count and Prognosis in Patients with Sepsis

The enrolled 206 patients were divided into two groups, decreased group and nondecreased group, based on different lymphocyte counts. We compared age, length of ICU stay, APACHE II score, SOFA score, proportion of shock occurrence, ICU mortality, 28-day mortality, and 1-year mortality between the two groups to assess their differences. Results showed that patients with decreased CD3^+^, CD4^+^, and CD8^+^ counts were older than those with nondecreased CD3^+^, CD4^+^, and CD8^+^ counts and the difference was statistically significant (*P* < 0.05 for all). However, no significant differences were found in ICU mortality, 28-day mortality, 1-year mortality, and incidence rate of shock. Patients with decreased CD3^+^ counts had longer hospital stays and higher mechanical ventilation rates than those with nondecreased CD3^+^ counts. The APACHE II score was higher in the group of decreased CD3^+^ and CD8^+^ counts (*P* < 0.05 for all). The 28-day mortality rate was higher in the CD4^+^/CD8^+^ decreased group than in the nondecreased group, but the difference was not statistically significant (*P*=0.29), and no differences were observed in age, length of ICU stay, APACHE II score, SOFA score, proportion of shock occurrence, ICU mortality, and 1-year mortality (Table 3).

We compared the mechanical ventilation patients with different CD3^+^, CD4^+^, and CD8^+^ count groups and the incidence of ventilator-associated pneumonia, as well as different CD4^+^/CD8^+^ ratio groups, following the above grouping principles. Results showed that the mechanical ventilation duration was longer in the group with decreased CD3^+^ counts group compared with the nondecreased group. There were statistically significant differences between 144 h (48, 360) and 96 h (48, 144), respectively (*P* < 0.05), while there was no significant difference in the incidence of ventilator-associated pneumonia (Table 4).

To identify risk factors affecting the mortality rate at 28 days, we performed multivariate logistic regression analysis. Baseline variables that were considered clinically relevant with outcome entered into multivariate logistic. Furthermore, the age was excluded due to collinearity with the APACHE II score. The CD4^+^ and CD8^+^ counts were excluded due to collinearity with the CD3^+^ count. Variables for inclusion were carefully chosen. As a result, gender, APACHE II score, platelet, procalcitonin, CD3^+^ count, and CD4^+^/CD8^+^ ratio were entered into multivariate logistic (Figure S1). Results showed that only the APACHE II score was correlated with the 28-day mortality (Table S1).

**Figure 1:**
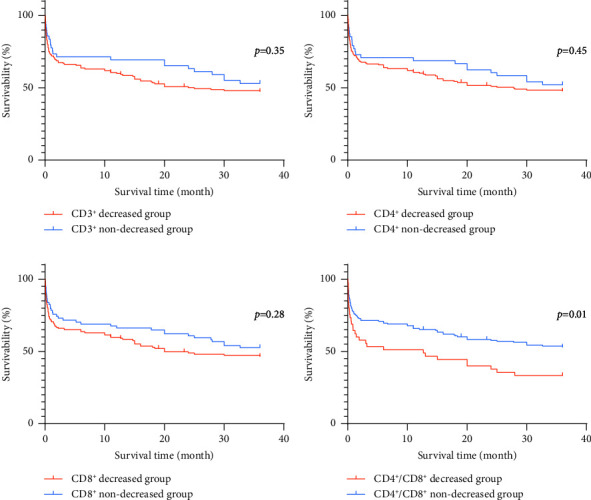
Survival curves of different subgroups of lymphocyte subtypes. (a) CD3^+^ survival curve; (b) CD4^+^ survival curve; (c) CD8^+^ survival curve; (d) CD4^+^/CD8^+^ survival curve.

Three years after discharge, patients with sepsis in the above groups were followed up, and Kaplan–Meier survival curves were plotted. Log-rank test was then performed to compare these curves. Results showed that there were no significant differences in the survival curves of CD3^+^, CD4^+^, and CD8^+^ groups (*P* > 0.05). In contrast, the survival curve of the the CD4^+^/CD8^+^ ratio group showed a statistically significant difference (*P*=0.01), and the survival rate of the CD4^+^/CD8^+^ decreased group was lower (Figure 1).

## 4. Discussion

Many experiments and evidence-based medical research have confirmed that when pathogens invade the body and cause infection, they trigger a two-phase systemic immune response: the cytokine storm/systemic inflammatory response syndrome (SIRS) and immune suppression. In order to maintain individual survival and internal environment stability, the body initiates a series of negative feedback mechanisms such as neural and humoral pathways, leading to the occurrence of immune suppression [18, 19]. These two phases are present throughout the course of sepsis and can occur sequentially or in combination [20]. Pathological studies from clinical autopsies have shown increased immune cell apoptosis and increased proportions or expression of immune-suppressive cells or receptors, all of which are involved in the occurrence and/or development of sepsis-induced immune suppression [21]. Research by a Chinese professor has demonstrated that immunomodulatory peptide thymosin alpha 1 can improve the immunosuppressive state of severe sepsis [22]. However, sepsis is considered an unbalanced immune response, in which the pathogen evades immune defense mechanisms and continues to proliferate, leading to sustained stimulation and damage of host cells and an inability to restore internal balance. In this imbalanced reaction, many mechanisms that were initially activated to provide protection have become harmful [23]. Therefore, immunomodulatory therapy may become an effective means to reduce sepsis mortality. However, inappropriate timing of immunostimulant usage might have adverse effects. So, it is crucial to further clarify the characteristics of immune status changes in sepsis patients to select appropriate intervention timing and improve their prognosis [24].

The detection of immune function status in sepsis patients involves both the innate and acquired immune systems. Immunosuppression can be manifested as immune cell exhaustion. Since T lymphocytes are the most functionally robust and abundant immune cells and the primary effectors of anti-infection immune responses, the most affected subset is T lymphocytes [25]. According to the 2020 expert consensus on the diagnosis and treatment of immunosuppression in sepsis, acquired immune dysfunction in sepsis can be manifested as a decrease in T-cell numbers and abnormal changes in T-cell subsets [26]. Examining T-cell subtype proportions and surface molecule levels can effectively reflect the immune status of sepsis patients and is one of the most easily obtainable indicators at the primary care level [27]. Boomer et al. [15] found that CD4^+^ and CD8^+^ expression levels of septic patients were significantly decreased compared to those of nonseptic patients. Recent studies have also shown that subsets of lymphocytes, such as CD3^+^ T cells and CD4^+^ T cells, are independent risk factors for ICU-acquired infections [28]. This study showed that most of the enrolled sepsis patients had decreased CD3^+^, CD4^+^, and CD8^+^ cell counts within 24 hours of ICU admission, which is consistent with the findings of Cui et al. [29]. This suggests that the early decrease in T lymphocyte count in patients with sepsis may be one of the manifestations of secondary immunosuppression.

CD3^+^ is expressed on the surface of all mature T cells and can reflect the overall immune function status of the body. Based on the differential expression of other CD molecules on their surface, T lymphocytes can be divided into two subsets: CD4^+^ and CD8^+^. The results showed that the CD3+, CD4+, and CD8+ counts in the decreased group had a significantly higher age compared to the CD3+, CD4+, and CD8+ counts in the nondecreased group. This may be due to the decreased proliferation capacity of hematopoietic stem cells in the bone marrow and thymic degeneration with increasing age, leading to physiological apoptosis of circulating immune cells due to progressive telomere shortening and ultimately resulting in age-related decline in the physiological immune function [30, 31]. Some scholars refer to this phenomenon as immune senescence [32, 33]. Moreover, the APACHE II score was significantly higher in the CD3+ and CD8+ count decreased group than in the nondecreased group, suggesting that the reduction in CD3+ and CD8+ T-cell counts might be associated with the severity of the disease in sepsis patients.

Immune cell exhaustion can increase susceptibility to opportunistic nosocomial infections and reactivation of latent viruses [34]. In 2012, Gentile et al. [35] found that survivors often experienced a series of clinical syndromes after early resuscitation and organ support. They proposed the concept of persistent inflammation immunosuppression catabolism syndrome (PICS), which is systematically summarized by prolonged hospital stays, persistent inflammatory response, immune suppression, and high protein catabolism, with high mortality. It is currently believed that bone marrow-derived suppressive cells are the main mechanism leading to severe PICS [36]. The results of this study showed that the CD3^+^ T lymphocyte count decreased group had a longer hospital stay and increased proportion and duration of mechanical ventilation compared to the nondecreased group, which suggests that immune dysfunction or paralysis may increase the risk of poor prognosis in patients. This study did not find a correlation between the decrease in CD3^+^, CD4^+^, CD8^+^T-cell counts, CD4^+^/CD8^+^ ratio, and the incidence of ventilator-associated pneumonia, which may be related to the small sample size of enrolled patients and the low number of cases of ventilator-associated pneumonia. Furthermore, large-scale clinical studies might need for verification.

CD4^+^ T lymphocytes can activate B cells and effector T cells by releasing various factors, thereby upregulating the body's immune function, while CD8^+^ T lymphocytes have surveillance and cytotoxic functions. The numbers and relative proportions of these lymphocyte subpopulations are in a relatively dynamic balance, mutually inducing and restraining each other, not only to clear foreign substances and resist infections but also to ensure that the body's own tissues are not damaged. When pathogens such as viruses and bacteria invade the body, this balance is disrupted, leading to decreased immune function. Therefore, the CD4^+^/CD8^+^ ratio is one of the important indicators reflecting the cellular immune status [37, 37]. The decrease of CD4^+^/CD8^+^ is closely related to immune suppression and poor prognosis [38], and improving the CD4^+^/CD8^+^T-cell ratio through intervention treatment can improve patient outcomes [39]. The results of this study showed that the group with decreased CD4^+^/CD8^+^ had a higher 28-day mortality compared to the nondecreased group; however, the difference did not reach statistical significance. Furthermore, the results of multifactorial logistic regression analysis also did not find the decrease in CD4+/CD8+ was associated with an increase in 28-day mortality. Further research may be needed to clarify this.

With advancements in critical care medicine and goal-directed interventions, the early mortality of sepsis patients has decreased. However, the rates of readmission, subsequently persistent, recurrent hospital-acquired, secondary infections, and elevated long-term mortality have continued to rise after the acute event “recovery” [6]. An increasing number of researchers have shifted their focus to the relationship between alterations in cellular immune function and the long-term prognosis of sepsis [8]. Several reports suggest that advanced age, comorbidities, and the sustained and simultaneous presence of inflammation and anti-inflammation states induce sustained immune dysfunction, immunosuppression, metabolic wasting, and inflammation [15, 35, 40, 41]. Immunocytes undergo varying degrees of functional changes within the first 48 hours after the onset of sepsis, and the occurrence of immune suppression during this period is closely associated with sustained multiple organ dysfunctions and poor prognosis [42]. In addition, persistent inflammation, prolonged immobilization, and catabolic and paralytic medications contribute to immune dysregulation, further promoting the occurrence of infectious complications, chronic deterioration, and death [43]. Therefore, researchers have been compelled to refocus their efforts on studying the impact of underlying immune system dysregulation on the development of infectious complications, sepsis recovery, and long-term mortality [44]. Existing research has demonstrated that patients are frequently rehospitalized in the 90 days after severe sepsis and the early reduction of innate immune cells in sepsis patients can lead to a persistent state of immune suppression, which predicts one-year mortality [45, 46]. This study followed up on 206 sepsis patients for three years after discharge, calculated survival rates, further plotted Kaplan–Meier survival curves, and conducted log-rank tests. The results showed that the CD4^+^/CD8^+^ decreased group had a lower survival rate compared with the nondecreased group, while there was no difference between different groups of CD3^+^, CD4^+^, and CD8^+^ counts. This suggests that the counts of CD3^+^, CD4^+^, and CD8^+^ decreased group are not significantly related to long-term prognosis. However, the decreased CD4^+^/CD8^+^ ratio was correlated with long-term survival rate decline. Nevertheless, large-scale studies might still need to further confirm these findings.

This study aimed to investigate the early detection of CD3^+^, CD4^+^, and CD8^+^T-cell subsets in adult sepsis patients admitted to ICU and conducted a detailed analysis of both short-term and long-term prognostic indicators. However, there are certain limitations to this study. First, it was a retrospective observational study, and multiple measurements of cell subsets were not performed for enrolled patients to determine the dynamic changes of immune cell subsets as the disease progresses. This limited our ability to understand the characteristics of immune cell subset alterations during the course of the disease. Second, no specific clinical interventions targeting immunity were performed, and the improvement of immune cell subpopulations and their impact on prognosis could not be clearly determined. This is also part of our plan for future research.

In conclusion, this study found that most sepsis patients might have decreased CD3^+^, CD4^+^, and CD8^+^ T lymphocyte counts in the early stages, and the decrease in CD3^+^ T lymphocytes and the decline in the CD4^+^/CD8^+^ ratio are correlated with the poor prognosis of sepsis patients. These parameters can serve as effective indicators for monitoring the early immune status of sepsis patients in primary healthcare facilities, providing a basis for immunomodulatory therapy in the management of sepsis.

## Figures and Tables

**Table 1:** General characteristics of included patients.

General characteristics	Number (*n* = 206)
Male, *n* (%)	130 (63.1%)
Age, years, mean ± SD	62.13 ± 16.06
APACHE II score, mean ± SD	16.01 ± 6.50
SOFA score, median, [IQR]	6.00 (4.00, 9.00)
Infection site *n* (%)	
Lungs	117 (56.0%)
Abdomen	87 (42.2%)
Urinary tract	16 (7.7%)
Skin and soft tissue	19 (9.2%)
Bloodstream	20 (9.7%)
Intracranial	2 (0.9%)
ICU mortality, *n* (%)	35 (16.9%)
Length of ICU stay, days, median [IQR]	8.00 (4.00, 15.00)

**Table 2:** Changes in T-cell counts of included sepsis patients (*n* = 206).

	Decreased (*n*, %)	Normal (*n*, %)	Increased (*n*, %)
CD3^+^ count	158 (76.7%)	42 (20.4%)	6 (2.9%)
CD4^+^ count	158 (76.7%)	42 (20.4%)	6 (2.9%)
CD8^+^ count	131 (63.6%)	71 (34.5%)	4 (1.9%)
CD4^+^/CD8^+^ ratio	45 (21.8%)	142 (68.9%)	19 (9.3%)

**Table 3:** Prognostic comparison between different T-cell count groups in sepsis patients.

	CD3^+^T-cell counts			CD4^+^T-cell counts			CD8^+^T-cell counts			CD4^+^/CD8^+^ ratio		
	CD3^+^ decreased group (*n* = 157)	CD3^+^ nondecreased group (*n* = 49)	*P* value	CD4^+^ decreased group (*n* = 158)	CD4^+^ nondecreased group (*n* = 48)	*P* value	CD8^+^ decreased group (*n* = 131)	CD8^+^ nondecreased group (*n* = 75)	*P* value	CD4^+^/CD8^+^ nondecreased group (*n* = 161)	CD4^+^/CD8^+^ decreased group (*n* = 45)	*P* value
Age, mean ± SD, years	63.53 ± 15.82	57.61 ± 16.15	0.02	63.73 ± 15.62	56.85 ± 16.53	0.01	64.06 ± 15.64	58.75 ± 16.33	0.02	62.18 ± 15.44	61.91 ± 18.30	0.91
Length of ICU stay, median, [IQR], d	11.00 (4.5, 21.0)	7.00 (4.0, 17.0)	0.03	8 (4, 14)	8.00 (4.00, 13.0)	0.88	7 (4.0, 14.0)	9.00 (5.0, 18.0)	0.12	8.0 (4.0, 15.0)	8.0 (4.0, 19.0)	0.69
APACHE II score, mean ± SD	16.90 ± 6.83	14.39 ± 5.62	0.01	16.72 ± 6.65	14.92 ± 6.47	0.10	17.05 ± 6.61	15.00 ± 6.51	0.03	16.31 ± 6.65	16.27 ± 6.65	0.96
SOFA score, median, [IQR]	7.00 (4.00, 9.00)	6.00 (4.00, 9.00)	0.88	6.00 (4.00, 10.00)	6.00 (3.00, 9.00)	0.13	6.00 (4.00, 9.00)	6.00 (4.00, 10.00)	0.92	6.00 (4.00, 9.00)	7.00 (5.00, 10.00)	0.11
Shock, *n* (%)	82 (52.22%)	22 (44.89%)	0.73	81 (51.27%)	23 (47.92%)	0.74	60 (45.8%)	44 (58.7%)	0.08	79 (49.1%)	25 (55.6%)	0.50
MV, *n* (%)	102 (67.5%)	25 (51.0%)	0.04	96 (61.39%)	31 (64.58%)	0.73	81 (61.8%)	46 (61.3%)	1.0	63 (60.9%)	29 (64.4%)	0.73
ICU mortality, *n* (%)	29 (18.47%)	6 (12.24%)	0.34	25 (15.82%)	10 (20.83%)	0.84	26 (19.8%)	9 (12.0%)	0.18	27 (16.8%)	8 (17.8%)	0.82
28-day mortality, *n* (%)	45 (28.66%)	11 (22.45%)	0.39	43 (27.21%)	13 (27.08%)	0.99	40 (30.5%)	16 (45.7%)	0.11	41 (25.5%)	15 (33.3%)	0.29
1-year mortality, *n* (%)	65 (41.4%)	17 (34.7%)	0.50	63 (39.9%)	19 (39.6%)	0.97	53 (40.5%)	29 (38.7%)	0.88	59 (36.6%)	23 (51.1%)	0.09
3-year survival, *n* (%)	76 (47.8%)	26 (53.1%)	0.62	76 (48.1%)	25 (52.1%)	0.38	62 (47.3%)	39 (52.0%)	0.31	86 (53.4%)	15 (33.3%)	0.01

SD, standard deviation; APACHE, acute physiology and chronic health evaluation; IQR, interquartile range; MV, mechanical ventilation.

**Table 4:** Comparison of different T-cell counts in septic patients undergoing mechanical ventilation.

	CD3^+^ T cells			CD4^+^ T cells			CD8^+^ T cells			CD4^+^/CD8^+^ ratio		
	CD3^+^ decreased group (*n* = 91)	CD3^+^ nondecreased group (*n* = 36)	*p* value	CD4^+^ decreased group (*n* = 95)	CD4^+^ nondecreased group (*n* = 32)	*p* value	CD8^+^ decreased group (*n* = 81)	CD8^+^ nondecreased group (*n* = 46)	*p* value	CD4^+^/CD8^+^ decreased group (*n* = 54)	CD4^+^/CD8^+^ nondecreased (*n* = 73)	*p* value
MV time, h, median, [IQR]	144 (48, 360)	96 (48, 144)	0.04	72 (36, 168)	96 (48, 204)	0.32	72 (36, 180)	84 (48, 150)	0.69	84 (48, 168)	72 (42, 168)	0.75
VAP, *n* (%)	10 (10.9%)	3 (8.33%)	0.76	10 (10.9%)	3 (8.33%)	0.76	11.1 (10.9%)	4 (8.7%)	0.90	5 (9.3%)	8 (11.0%)	0.76

MV, mechanical ventilation; VAP, ventilator-associated pneumonia.

## Data Availability

The data supporting the findings of this study are available upon request from the corresponding author.

## Supplementary Materials

Supplementary Materials Supplementary Table 1: logistic regression analysis of risk factors for 28-day mortality in septic patients. Supplementary Figure 1: correlation of the variables in multivariate analysis.
