# Supplementary material for: Changes in Early T-Cell Subsets and Their Impact on Prognosis in Patients with Sepsis: A Single-Center Retrospective Study
Source: Int J Clin Pract. 2023 Dec 26;2023:1688385. doi: 10.1155/2023/1688385 (PMC10761232; doi:10.1155/2023/1688385)
Supplement: Supplementary Materials — Supplementary Table 1: logistic regression analysis of risk factors for 28-day mortality in septic patients. Supplementary Figure 1: correlation of the variables in multivariate analysis. [file 1688385.f1.docx]

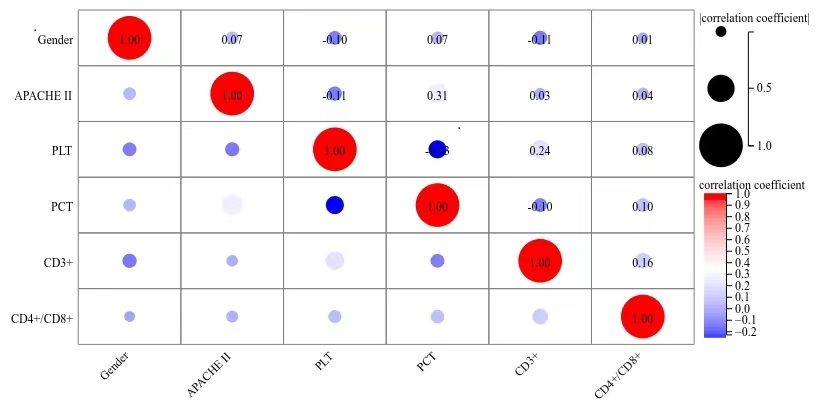


Figure S1. Correlation of the variables in multivariate analysis .

| **Table S1** **Logistic Regression Analysis of Risk Factors for 28-day Mortality in Septic Patients** | | | |
| --- | --- | --- | --- |
|  | OR | 95% CI | p-value |
| APACHE II Score | 1.10 | 1.04-1.16 | 0.001 |
| Gender | 1.82 | 0.86-3.89 | 0.12 |
| CD3^+^ | 1.00 | 1.00-1.01 | 0.57 |
| CD4^+^/CD8^+^ ratio | 0.515 | 0.23-1.16 | 0.11 |
| procalcitonin | 1.01 | 0.99-1.01 | 0.44 |
| platelet | 1.01 | 0.99-1.01 | 0.59 |
